# Effects of Fatty Acids on Benzo[a]pyrene Uptake and Metabolism in Human Lung Adenocarcinoma A549 Cells

**DOI:** 10.1371/journal.pone.0090908

**Published:** 2014-03-20

**Authors:** Rola Barhoumi, Youssef Mouneimne, Robert S. Chapkin, Robert C. Burghardt

**Affiliations:** 1 Department of Veterinary Integrative Biosciences, Texas A&M University, College Station, Texas, United States of America; 2 KAS CRSL, American University of Beirut, Beirut, Lebanon; 3 Department of Nutrition and Food Science, Texas A&M University, College Station, Texas, United States of America; Max Delbrueck Center for Molecular Medicine, Germany

## Abstract

Dietary supplementation with natural chemoprotective agents is receiving considerable attention because of health benefits and lack of toxicity. In recent in vivo and in vitro experimental studies, diets rich in n-3 polyunsaturated fatty acids have been shown to provide significant anti-tumor action. In this investigation, the effects of control fatty acids (oleic acid (OA), linoleic acid (LA)) and n-3 PUFA, e.g., docosahexaenoic acid (DHA) on the uptake and metabolism of the carcinogenic polycyclic aromatic hydrocarbon, benzo[a]pyrene (BaP) was investigated in A549 cells, a human adenocarcinoma alveolar basal epithelial cell line. A549 cells activate BaP through the cytochrome P450 enzyme system to form reactive metabolites, a few of which covalently bind to DNA and proteins. Therefore, multiphoton microscopy spectral analysis combined with linear unmixing was used to identify the parent compound and BaP metabolites formed in cells, in the presence and absence of fatty acids. The relative abundance of select metabolites was associated with altered P450 activity as determined using ethoxyresorufin-O-deethylase activity in cells cultured in the presence of BSA-conjugated fatty acids. In addition, the parent compound within cellular membranes increases significantly in the presence of each of the fatty acids, with the greatest accumulation observed following DHA treatment. DHA treated cells exhibit significantly lower pyrene-like metabolites indicative of lower adducts including DNA adducts compared to control BSA, OA or LA treated cells. Further, DHA reduced the abundance of the proximate carcinogen BaP 7,8-dihydrodiol and the 3-hydroxybenzo[a]pyene metabolites compared to other treatments. The significant changes in BaP metabolites in DHA treated cells may be mediated by the effects on the physicochemical properties of the membrane known to affect enzyme activity related to phase I and phase II metabolism. In summary, DHA is a highly bioactive chemo-protective agent capable of modulating BaP-induced DNA adducts.

## Introduction

Exposure to polycyclic aromatic hydrocarbons (PAHs) usually occurs by breathing contaminated air or by eating grilled foods. Several PAHs have been listed by the U.S. Environmental Protection Agency as probable human carcinogens and this includes the prototype carcinogenic PAH, benzo[a]pyrene (BaP) [1]. As a ligand for the aryl hydrocarbon receptor (AhR), BaP upregulates the expression of phase I bioactivation genes and phase II conjugation genes [2], [3]. Induction of biotransformation enzymes including CYP1A1, CYP1B1 and epoxide hydrolase metabolically activate BaP to different types of metabolites including hydroxylated intermediates, epoxides, quinones, dihydrodiols, dihydrodiol epoxides and various metabolite-conjugates in cells [4], [5]. BaP toxicity results from the bioactivation of BaP to the ultimate toxic compound, benzo[*a*]pyrene-7,8-dihydrodiol-9,10-epoxide (BPDE). BPDE can alkylate DNA to form BPDE-DNA adducts (BPDE-N(2)-deoxyguanosine [BPDE-dG]), which have been associated with BaP-induced carcinogenesis [6]. In addition, many of the major metabolites can be conjugated to glucuronic acid, sulfate and glutathione to become more water soluble facilitating excretion [7].

Studies over the last four decades have shown that diet can modulate the response of organisms to drug absorption, distribution, metabolism, and excretion [8], [9]. Diet is also known to modulate carcinogenesis; for example, n-3 fatty acids, especially the long-chain polyunsaturated fatty acids (PUFAs), eicosapentaenoic acid (EPA) and docosahexaenoic acid (DHA) present in fatty fish and fish oils inhibit carcinogenesis [10], [11]. Furthermore, dietary fish oil has recently been shown to play a protective role in PAH- (including BaP-) induced hepatic carcinogenesis and to significantly reduce levels of DNA adducts in mice, leading to further reinforcement of the idea that fish oil can be used as a cancer chemopreventative agent [12].

In a previous work, we demonstrated that with the use of the multiphoton microscopy imaging at an excitation of 740 nm and the mathematical unmixing process, it is possible to simultaneously identify 8 of the major metabolites of BaP in real time in live cultured cells. This technique proved useful to quantify specific metabolites in [13].

Therefore, the aims of this study were: (1) to investigate the role of some dietary fatty acids (polyunsaturated fatty acids: n-3 PUFAs (DHA), n-6 PUFA linoleic acid (LA), n-9 monounsaturated fatty acid [n-MUFA] or oleic acid [(OA])) in BaP metabolic activation and metabolism in A549 lung adenocarcinoma cells using the spectral unmixing approach and (2) to elucidate how these dietary factors may provide increased or decreased protection against BaP-induced adduct formation.

## Materials and Methods

### Materials

Dulbecco's Modified Eagle's Medium/Ham's Nutrient Mixture F-12 (DMEM-F12), fatty acid free bovine serum albumin (BSA), Dulbecco's phosphate buffered saline (PBS), glutathione (GSH), benzo[a]pyrene (BaP), L- buthionine sulfoximine (BSO), janus green, β-glucuronidase (keyhole limpet, #G8132), triclosan (Irgasan), resorufin, ethyl ether, 3,3′-methylene-bis(4-hydroxycoumarin) (dicumarol), and trans-3,4′,-5-trihydroxystilebene; 3,4′,5-stilbenetriol (resveratrol) were purchased from Sigma-Aldrich Chemical Co. (St. Louis, MO). Fetal bovine serum (FBS) was obtained from Equitech-Bio (Kerrville, TX). 3-Hydroxybenzo[a]pyrene (3OH), 9-Hydroxybenzo[a]pyrene (9OH), benzo(a)pyrene-*trans*-7, 8- dihydrodiol(+/−) (t7,8), benzo[a]pyrene-r-7,*t*-8-dihydrodiol-*t*-9,10-epoxide(±),(*anti*) (BPDE), pyrene (Pyr), benzo(a)pyrene-3-sulfate (3-S), and benzo[a]pyrene-3,6-dione (3,6BPQ), were purchased from Midwest Research Institute (Kansas City, MO) which operates the Chemical Carcinogen Reference Standard Repository. Analytical data provided with each standard was reported as >99% pure by HPLC and UV/visible spectra. These UV/visible spectra were also confirmed prior to use in cells. Tissue culture flasks, 2-well Lab-Tek chambered coverglass slides were purchased from Thermo Fisher Scientific (Waltham, MA) and multi well plates were purchased from Corning Inc. (Corning, NY). The *XIT* Genomic DNA kit was purchased from G-Biosciences (St. Louis, MO). The OxiSelect BPDE DNA Adduct ELISA Kit was purchased from Cell Biolabs Inc. (San Diego, CA). Fatty acids (oleic acid (OA), linoleic acid (LA) and docosahexaenoic acid (DHA)) were purchased from NuChek (Elysian, MN) and were complexed with BSA to form aqueous-soluble reagent that can be absorbed and utilized by cells [14]. BaP, 3OH, t7,8 were each prepared as 1 mM stocks in DMSO. Resorufin ethyl ether was prepared as a 1 mM stock in methanol and diluted to 4 µM for EROD activity measurement. Janus green was prepared in PBS at 1 mg/ml. Both GSH and BSO were prepared as 10 mM stock in DMEM-F12.

Alpha-naphtoflavone (αNF) was purchased from Fisher Scientific (Pittsburg, PA) and prepared as 100 mM stock in DMSO. Monochlorobimane was purchased from Life Technologies (Grand island, NY) and pepaed as 50 mM stock in DMSO.

### Cell Culture

The A549 cell line, derived from type II pneumocytes (CCL 185) was obtained from American Type Culture Collection (Manassas, VA). A549 cells express phase I and II enzymes involved in detoxification or bioactivation of pulmonary toxicants and respond to P450 inducers, albeit at a lower level than normal human type II pneumocytes [15]. Cells were cultured in DMEM-F12 medium with 10% FBS. Cultures were approximately 80% confluent at the time of analysis.

### Ethoxyresorufin-O-deethylase (EROD) Activity

EROD activity is a biomarker of exposure to planar halogenated and polycyclic aromatic hydrocarbons (PHHs and PAHs, respectively) and provides evidence of aryl hydrocarbon receptor-mediated induction of cytochrome P450-dependent monooxygenases [16]. To quantify the induction of EROD activity following BaP and/or fatty acid treatments, cells were plated on 96 well-plates at 10,000/well for 24 h in the presence or absence of fatty acids prior to treatment with 0.5 µM BaP or vehicle control for another 48 h. Following two washes with PBS and three cycles of a freeze/thaw process (−80°C for 5 min), plates were then loaded with a mixture of 4 µM resorufin ethyl ether, 10 µM dicumarol and 0.5 mM NADPH for 30 min. EROD activity was measured using a BioTek Synergy 4 plate reader (Biotek Instruments Inc., Winooski, VT, USA) with an excitation wavelength of 560 nm and an emission wavelength of 590 nm. Cell number per well was determined using the Janus green assay (as described below) and EROD fluorescence intensities measured were normalized accordingly. Fifteen samples per treatment were collected and at least 3 experiments were performed on different days.

### Cell Count/Janus Green Assay

Following the EROD assay, Janus green (1 mg/ml) was added to each well and incubated at room temperature for 5 min. Cells were again washed twice with PBS and 100 µl of methanol was added to each well. Janus green signal was then measured using a BioTek Synergy 4 plate reader set to an absorbance of 630 nm.

### Multiphoton Spectral Analysis of BaP in A549 Cells

A549 cells were cultured for 48 h in DMEM F-12 with 10% FBS and 50 µM of each of the BSA-conjugated fatty acids (OA, LA or DHA) or BSA control on 2-well Lab-Tek slides at a density of 10^5^ cells per well. The dose of 50 µM of the fatty acids has been shown to alter cell signaling [17] and to induce no cytotoxicity in normal cells [18], [19]. Cells were then incubated for an additional 24 h with 2 µM BaP. Treatments were then removed by washing cells with serum- and phenol red-free culture medium and slides were transferred to the stage of a Zeiss 510 META NLO laser scanning microscope (Carl Zeiss Microimaging, Thornwood, NY). Spectral analysis of an area of 225×225 µm (typically containing 25 to 40 cells) was performed while irradiating cells with a Chameleon tunable Ti:Sapphire laser (Coherent Inc., Santa Clara, CA) at an excitation wavelength of 740 nm (which is roughly equivalent to 370 nm in single photon excitation with a continuous wavelength laser system). Using the lambda stack algorithm software available with the Zeiss 510 META NLO instrument, a fluorescence emission spectrum ranging from 399–600 nm with a 10.7 nm bandwidth was recorded for each scanned area. Thirty areas were scanned per treatment. All images were collected with a C-Apo 40×/1.2 NA water immersion objective designed for viewing specimens in an aqueous medium. BaP metabolites present in each treatment were then identified using the linear unmixing process based upon the use of a spectral database of BaP and metabolite standards generated under the same experimental conditions as previously described [13].

### Deciphering the Effects of Fatty Acids on BaP metabolism

To further determine the effects of each fatty acid on BaP metabolites generated in A549 cells, triclosan was used as a substrate and inhibitor of glucuronidation and sulfation [20]. In addition, β-glucuronidase was used as a catalyst for the hydrolysis of the water-soluble glucuronide conjugates of BaP generated in phase II detoxification reactions which is capable of increasing the hydroxyl metabolites of BaP [21]. To conduct the triclosan experiments, A549 cells were plated on coverglass slides and directly supplemented for 48 h with the fatty acid (OA, LA, or DHA) or BSA control followed by treatment with 2 µM BaP or the combination of 2 µM BaP and 40 µM triclosan for 24 h. Cells were then washed, scanned under the same experimental conditions and the t7,8 metabolites were evaluated. For β-glucuronidase experiments, cells were supplemented for 48 h with BSA-conjugated fatty acids (OA, LA or DHA) or BSA control followed by treatment with 1 µM 3OH, or a combination of 1 µM 3OH and 500 units/ml β-glucuronidase, 2 µM t7,8 or a combination of 2 µM t7,8 and 500 units/ml β-glucuronidase for 24 h. Cells were then washed, scanned on the stage of the multiphoton microscope and the 3OH and t7,8 metabolites were evaluated.

To further investigate the role of fatty acids in the metabolism of BaP through glutathionation, L-buthionine sulfoximine (BSO 1 mM-10 mM), a potent and specific inhibitor of glutathione (GSH) synthesis via γ-glutamyl synthase [22] and supplementation of cells with GSH were used. To determine the changes in metabolites generated by BaP due to treatments with BSO or GSH, cells were plated on coverglass slides in the presence of the BSA-conjugated fatty acids (OA, LA or DHA) or BSA control for 48 h followed by 2 µM BaP, 2 µM BaP and 1 mM BSO, or 2 µM BaP and 10 mM GSH for another 24 h. Cells were then washed and scanned on the stage of the multiphoton microscope and the 3OH, t7,8 and Pyr metabolites were evaluated.

To determine the changes in metabolites generated by 3OH due to treatments with BSO or GSH, cells were plated on coverglass slides in the presence of the BSA-conjugated fatty acids (OA, LA or DHA) or BSA control for 48 h followed by 1 µM 3OH, 1 µM 3OH and 1 mM BSO, or 1 µM 3OH and 10 mM GSH for another 24 h. Cells were then washed and scanned on the stage of the multiphoton microscope and the 3OH metabolites were evaluated.

### BPDE-DNA Adduct Measurements

DNA was isolated using the XIT Genomic DNA kit according to the manufacturer instructions. The spectrum of BPDE-DNA was identified from the BPDE-DNA standard provided in the OxiSelect™ BPDE DNA Adduct ELISA Kit. The BPDE-DNA signal was then determined with a Biotek Synergy plate reader using lysates of cells that were supplemented with 50 µM BSA-conjugated fatty acids (OA, LA or DHA) or BSA control for 48 h followed by 24 h of treatment with 2 µM BaP.

### Statistical Analysis

Measurements of EROD activity are presented as mean normalized fluorescence intensities ± S.E. of 15 samples per concentration and measurements of BPDE-DNA adducts are presented as means ± S.E. with 3 samples per treatment. Data collected by multiphoton microscopy are reported as mean fluorescence intensities ± S.E or normalized to the corresponding control of at least 30 images per treatment. All data were analyzed using two-sided t-tests for 2 samples comparison, One way- ANOVA followed by Tukey's test at P<0.05 for multiple treatment comparison, and two way ANOVA followed by Bonferroni's test at p<0.05 for combined time and treatment comparisons.

## Results

### Single Cell Multiphoton Spectral Analysis of BaP

**Figure 1:**
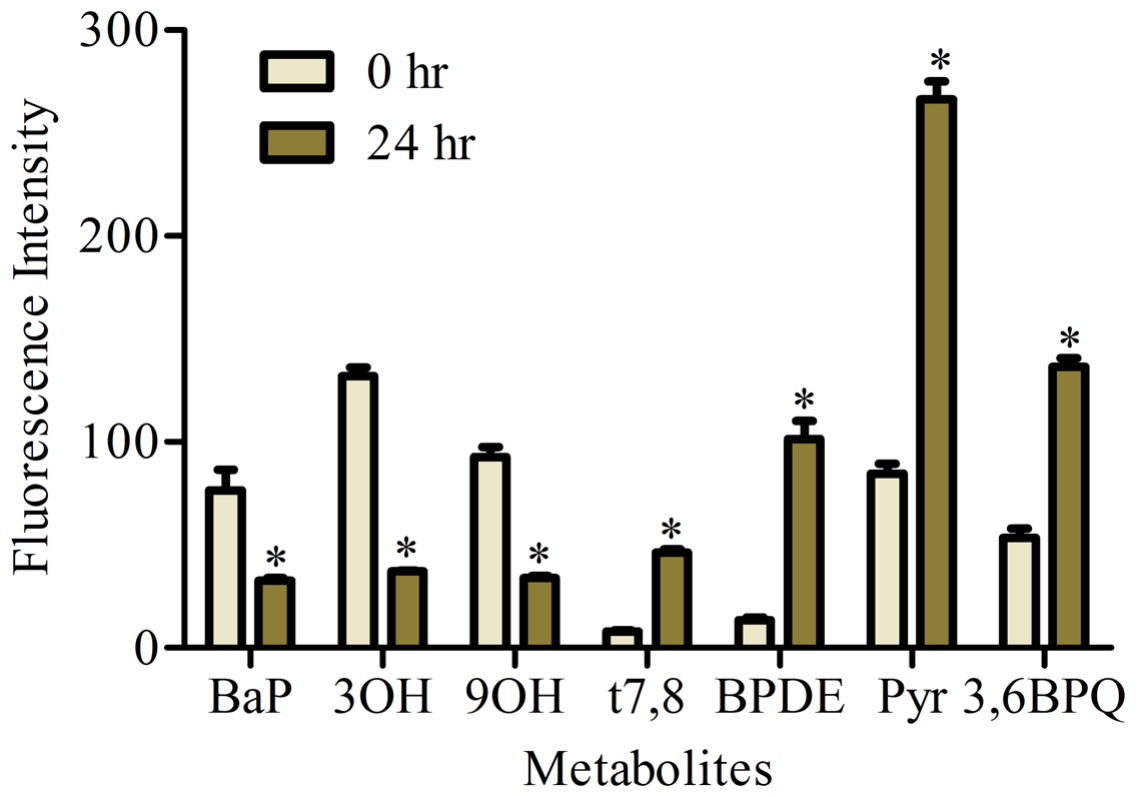
BaP uptake and metabolism in A549 cells. Cells were treated overnight with 2 µM BaP and metabolites were determined directly and 24 h following removal of BaP. Note the reduction in parent compound and the 3OH metabolite and the increase in 3,6,BPQ, t7,8, BPDE and pyrene-like metabolites (Pyr) 24 h after removal of BaP from the cultured cells. *indicates significant difference from the corresponding control metabolite at p<0.05.

A549 cells have been shown to have the capability to activate BaP through the cytochrome P450 enzyme system (**Figure A in File S1**) and to form reactive metabolites that covalently bind nucleophilic sites of cellular macromolecules such as DNA [23], [24]. Many of the BaP metabolites and their time-dependent changes in liver cells have been detected in living cells using multiphoton microscopy with the linear unmixing process [13]. Similar time-dependent changes in BaP levels and metabolites in A549 cells are shown in **Figure 1**. The parent compound and hydroxyl metabolites (3OH and 9OH) decreased significantly while the other reactive metabolites (t7,8, BPDE, Pyr and 3,6BPQ) increased significantly 24 h following removal of BaP. In addition, the AhR antagonist, α-naphtoflavone [25], [26] added simultaneously with BaP significantly reduced the Pyr and 3S metabolites (**Figure B in File S1**) while simultaneous treatment with BaP and resveratrol (another competitive antagonist of AhR ligands) [27] significantly reduced the parent compound BaP as well as the 9OH and Pyr metabolites (**Figure C in File S1**).

**Figure 2:**
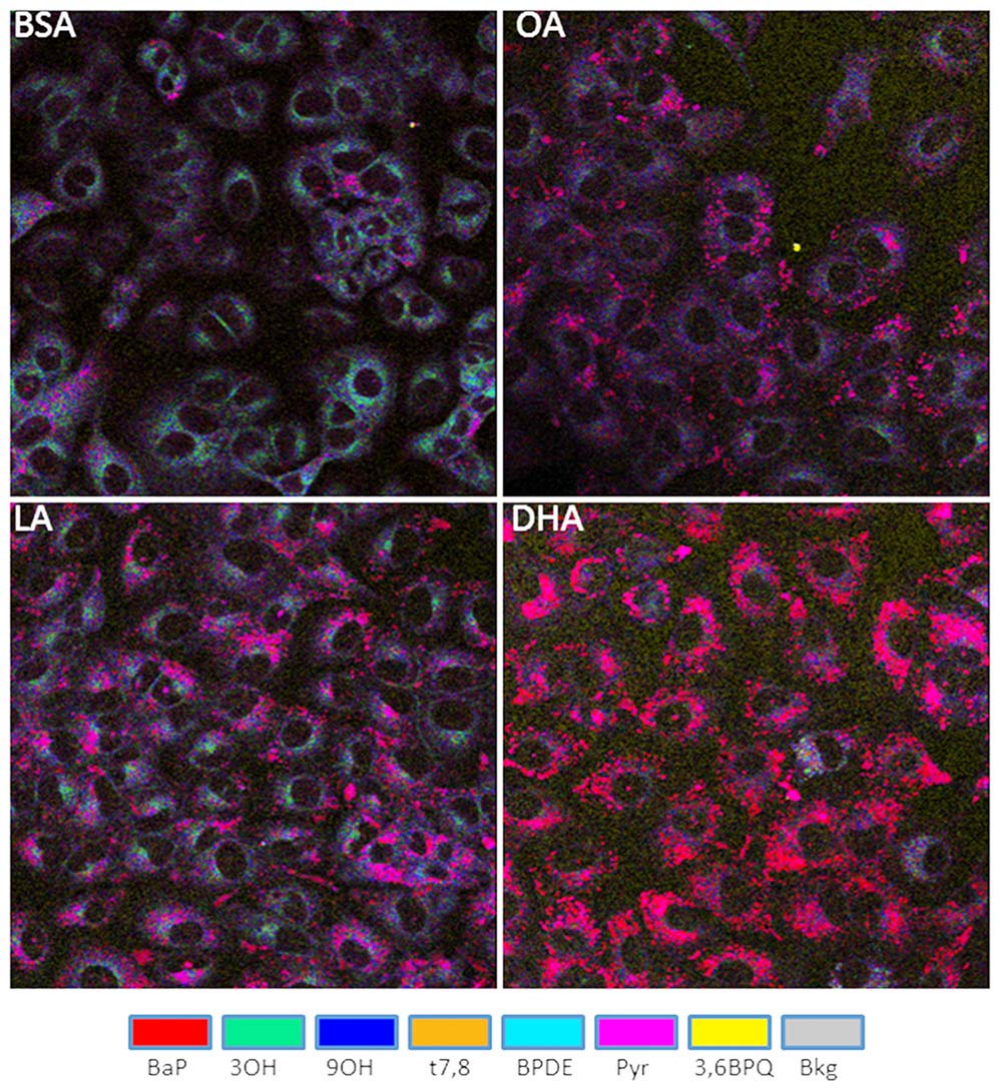
Pseudocolor images of BaP metabolites obtained with A549 cells supplemented with fatty acids. Cells were supplemented with each fatty acid: 50 µM BSA carrier control (top left panel), OA (top right panel), LA (bottom left panel), and DHA (bottom right panel). for 48 h prior to addition of 2 µM BaP for 24 h. Each image represents the overlay of 7 metabolites represented by referenced colors (BaP, 3OH, 9OH, t7,8, 3,6BPQ, BPDE and Pyr).

**Figure 3:**
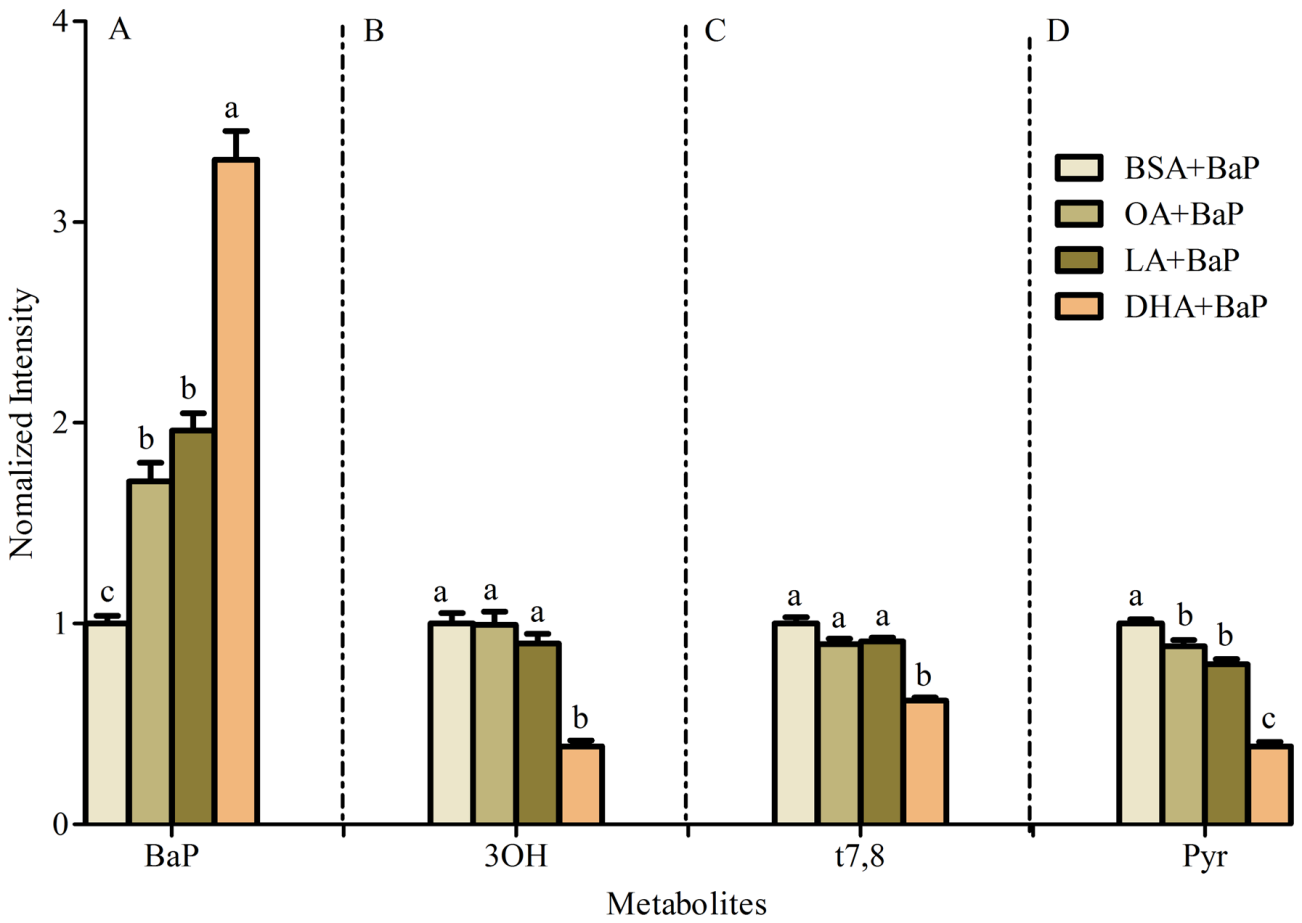
Significant BaP metabolites in A549 cells supplemented with fatty acids. BaP (A), 3OH (B), t7,8 (C) and Pyr (D) profiles in cells. A549 cells were incubated with BSA (carrier control), OA, LA or DHA for 48 h prior to addition of 2 µM BaP for 24 h. Data represent mean ± SEM of fluorescence intensity of significant metabolites: A) BaP, B) 3OH, C) t7,8, and D) Pyr measured in at least 30 images per treatment with at least 30 cells per image. Letters above the bars represent significant differences within a treatment for each evaluated metabolite using Tukey test at p<0.05. No significant differences were found in the other metabolites measured (9OH, BPDE or 3,BPQ).

**Figure 4:**
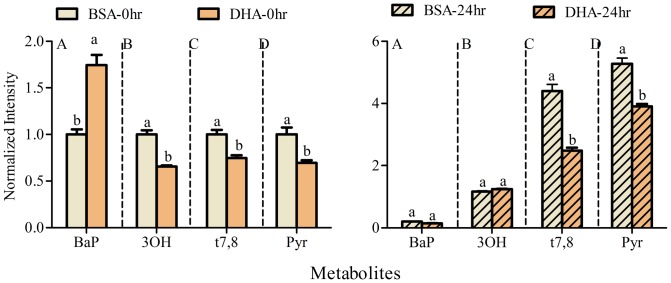
Comparison of BaP metabolites (BaP, 3OH, t7,8 and Pyr) in BSA and DHA supplemented cells. Cells were supplemented with BSA or DHA for 48 µM BaP for 24 h. Cells were then imaged directly 0 h (A) and 24 h (B) after removal of BaP. Note that the decrease in t7,8 and Pyr metabolites in DHA treated cells are persistent even 24 h after removal of BaP. Data represent mean normalized intensity (with respect to BSA 0 h) ± SEM of at least 30 images per treatment with at least 30 cells per image. Letters above the bars represent significant differences from the corresponding control for each evaluated metabolite using two-way ANOVA followed by Bonferroni test at p<0.05.

The relative abundance of several major metabolites changed when cells were cultured in the presence of BSA-conjugated fatty acids (OA, LA or DHA, each at 50 µM along with BSA as carrier control) for 24 h in advance of 2 µM BaP exposure for 24 h (**Figure 2**). Accumulation of the parent compound (BaP) within cellular membranes increased significantly in the presence of each of the three fatty acids (OA, LA, and DHA) when compared to control, with the greatest accumulation observed in DHA treated cells (**Figure 3A**). The 3OH and t7,8 metabolites were significantly lower only in DHA-treated A549 cells (**Figures 3B and 3C**). Furthermore, Pyr-like metabolites, thought to result from BPDE-adduct formation, decreased with each of fatty acids in the following order: DHA<LA = OA<BSA (**Figure 3D**). The significant changes in BaP metabolites (t7,8 and Pyr) obtained with DHA treatment persisted 24 h after removal of BaP and DHA (**Figure 4**). It is also noteworthy that the uptake of BaP up to 1 h is not significantly different between BSA and DHA treated cells (**Figure D in File S1**).

**Figure 5:**
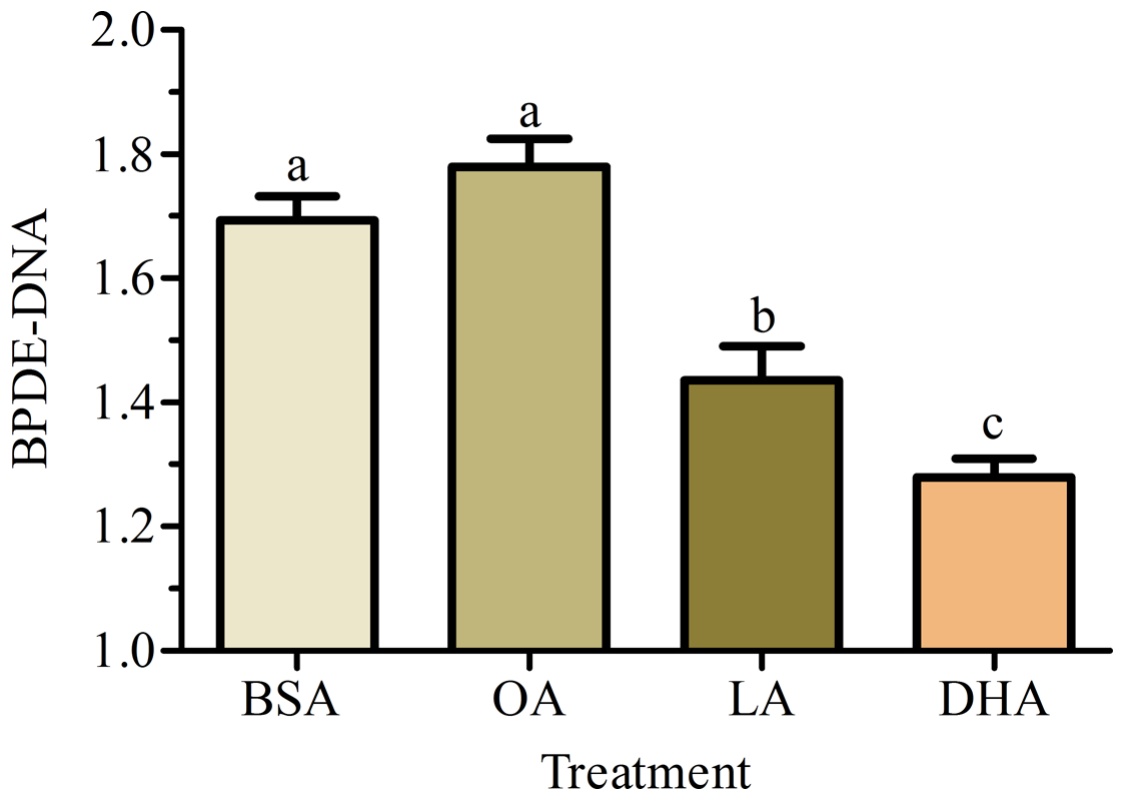
BPDE-DNA adduct formation in A549 cells supplemented with fatty acids. Cells were incubated with 50 µM OA, LA, DHA or BSA carrier for 48 h prior to addition of 2 µM BaP for 24 h. Values represent results from a typical experiment with mean ratio (BPDE-DNA/BPDE) ± SEM of at least 3 replicates per treatment. Different letter indicates significant difference using Tukey's multiple comparison test at p<0.05.

To confirm that the decrease in the Pyr-like signal with fatty acid treatments was due to reduced BPDE-DNA adducts, cells were supplemented with 50 µM OA, LA, DHA or BSA carrier for 48 h prior to addition of 2 µM BaP for 24 h. The cellular content of BPDE-DNA adducts was quantified. The emission of BPDE-DNA as a function of fatty acid and BaP exposure indicate that LA significantly decreased the BPDE-DNA adducts compared to BSA carrier while DHA induced the greatest reduction in the BPDE-DNA adducts (**Figure 5**).

### EROD Activity in BaP Treated A549 Cells

**Figure 6:**
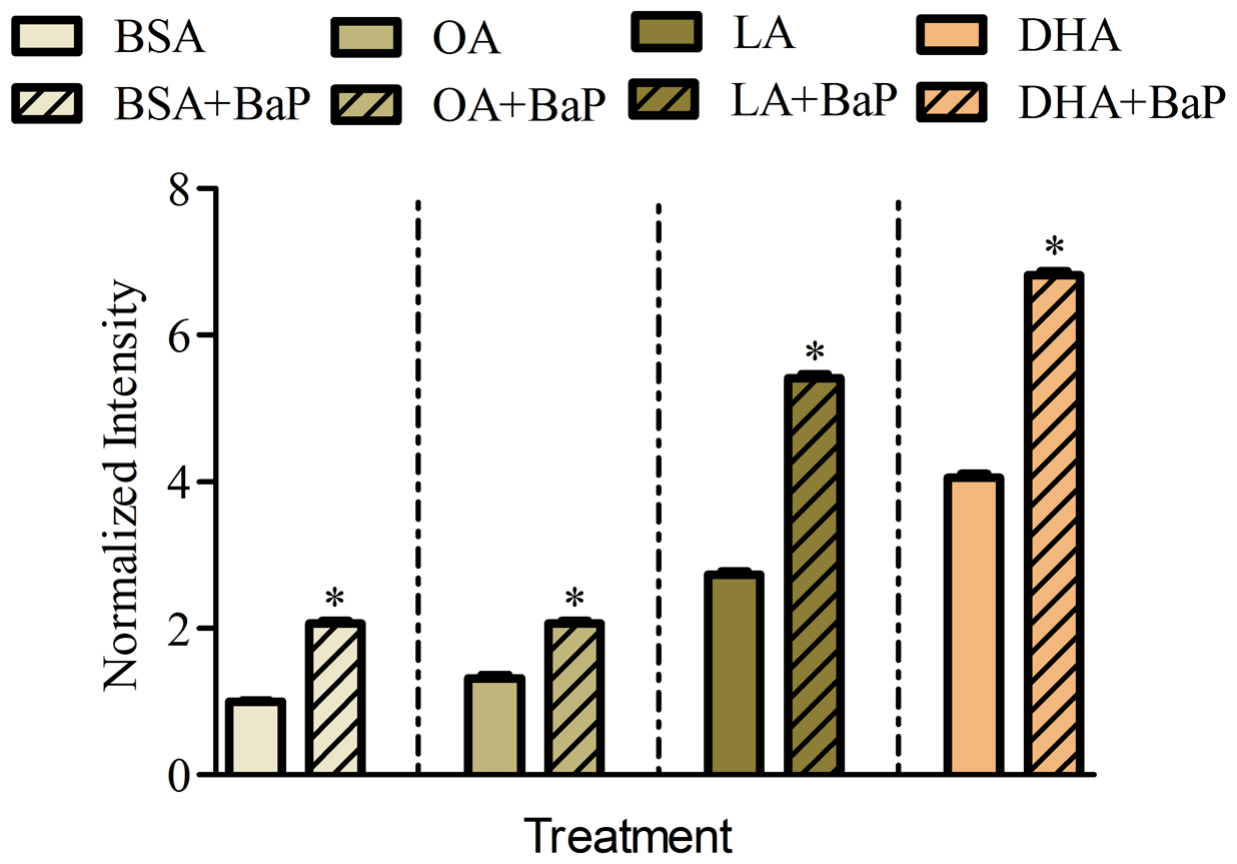
EROD activity in A549 cells supplemented with BSA-conjugated fatty acids (OA, LA, DHA, BSA). Cells were treated with each of the fatty acids for 48 µM BaP for 24 h. Data represent mean ± SEM of a typical experiment with 3 replicates per treatment. * indicates significant difference between two compared treatments using two-tailed t-test at p<0.05.

The effects of fatty acids on BaP activation of the cytochrome P450 enzyme system was confirmed by assessment of EROD activity in A549 cells supplemented with 50 µM BSA-conjugated fatty acids (OA, LA, DHA, BSA-carrier control) for 48 h followed by 0.5 µM BaP for 24 h (**Figure 6**). Cells treated with BSA+BaP exhibited a significant increase in EROD activity compared to basal EROD activity (BSA alone). OA alone did not significantly affect the basal EROD activity and the combination treatment of OA and BaP showed a similar EROD response to that of BSA+BaP. Both LA and DHA significantly enhanced EROD activity in the absence of BaP and further increased EROD activity in the presence of BaP, with DHA responses in the absence or presence of BaP significantly greater than LA.

### Fatty Acid Effects on Glutathionation in BaP Treated A549 Cells

**Figure 7:**
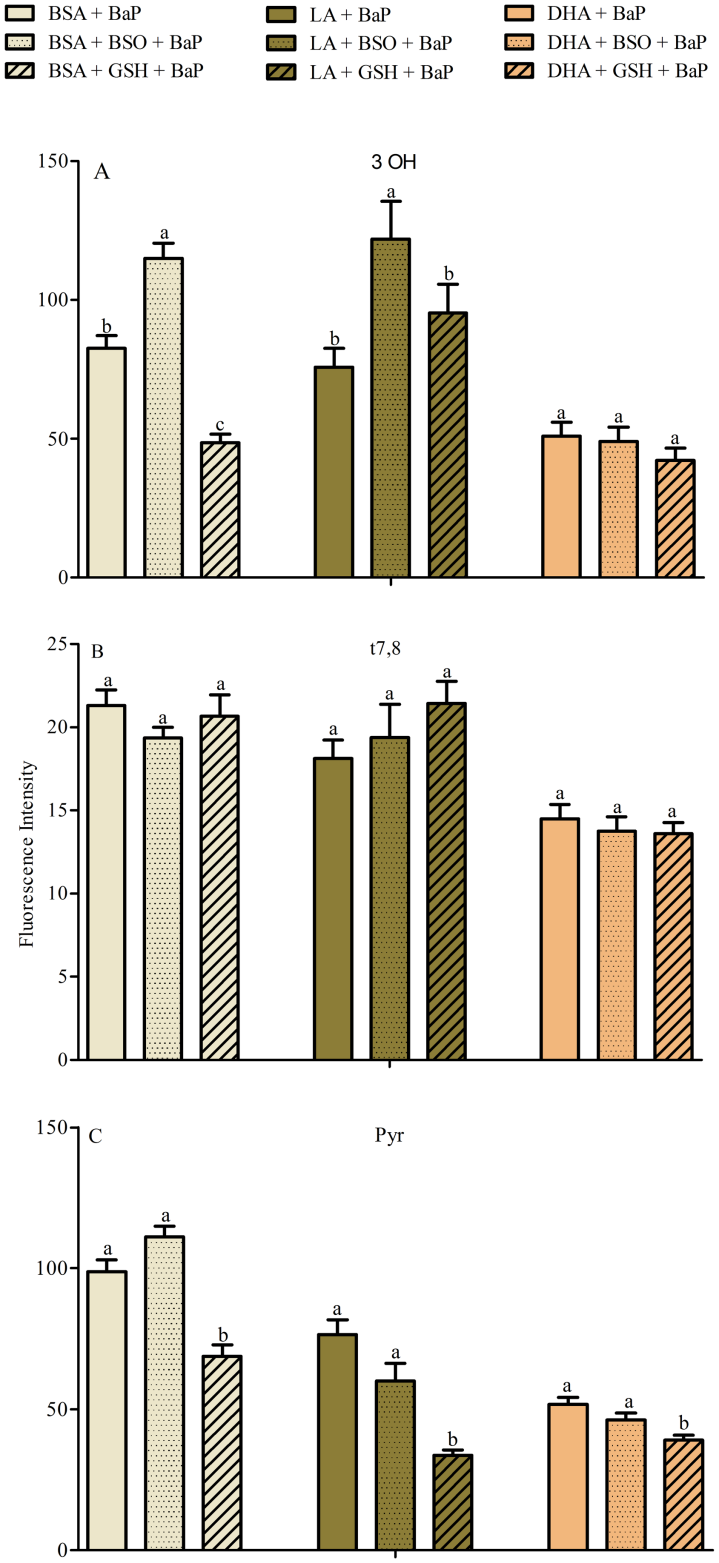
Glutathionation in A549 cells supplemented with BSA-conjugated fatty acids (LA, DHA, BSA carrier control). Cells were treated with BSO or GSH in combination with each of the fatty acids for 48 µM BaP for 24 h. Data represents mean normalized intensity ± SEM of at least 30 images per treatment and 30 cells per image. * indicates significant difference from the corresponding control using Tukey test at p<0.05. Note that DHA treated cells did not show any significant changes in the metabolites (3OH, t7,8 and Pyr) in the presence of BSO or GSH.

To investigate the role of fatty acids in the metabolism of BaP through glutathionation, L-buthionine sulfoximine and supplementation of cells with GSH were used. A549 cells supplemented with 50 µM BSA for 48 h followed by treatment with 2 µM BaP and 1 mM BSO (BSA+BSO+BaP) resulted in a significant increase in 3OH compared to BSA+BaP; and substitution of BSO by 10 mM GSH (BSA+GSH+BaP) resulted in a significant decrease in 3OH compared to BSA+BaP, presumably due to formation of glutathione conjugates [7]. BSO treatment induced similar results in LA treated cells while GSH had no significant effect (**Figure 7A**). DHA treatment alone reduced 3OH metabolites but addition of BSO or GSH to DHA did not cause any additional effects (**Figure 7A**). In addition, BSO or GSH did not alter t7,8 metabolites in any of the three treatments (BSA, LA or DHA) (**Figure 7B**). In addition, supplementation of cells with 10 mM GSH reduced the Pyr-like metabolites in BSA, LA and DHA treated cells (**Figure 7C**).

**Figure 8:**
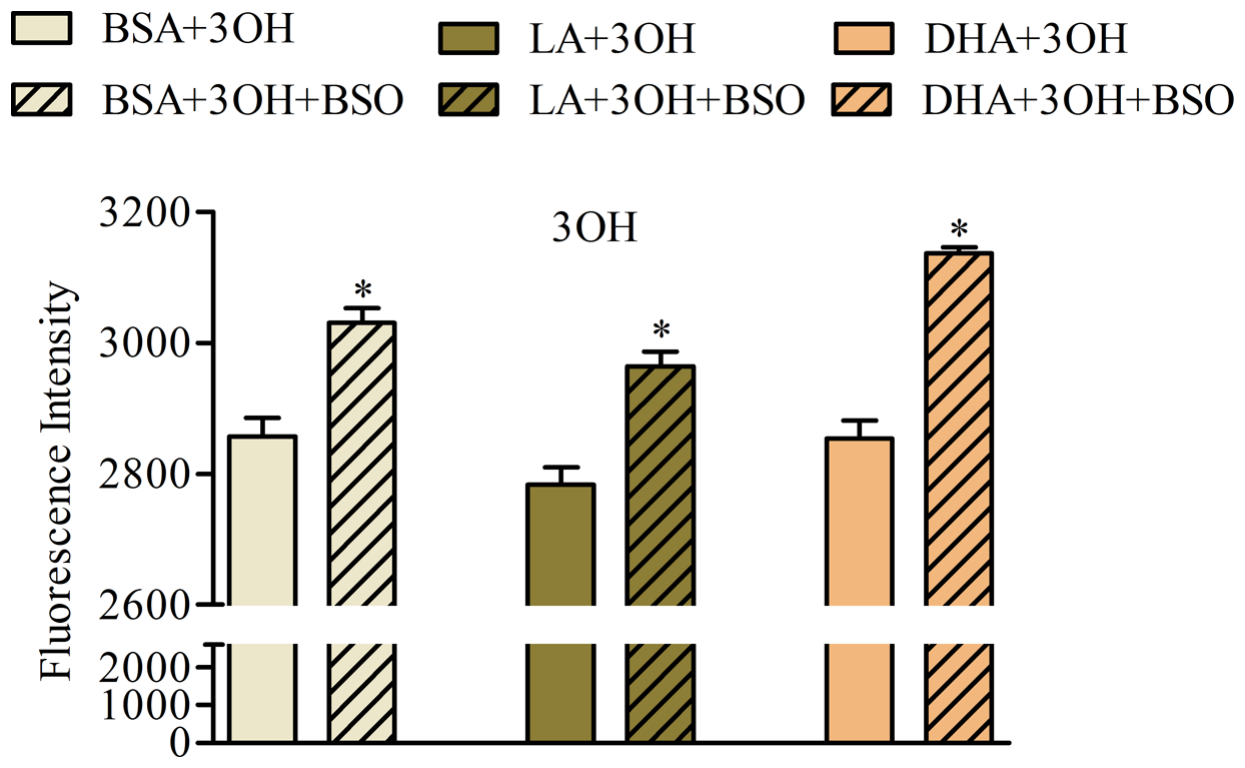
Effect of BSO on the glutathionation of 3OH in A549 cells supplemented with fatty acids. Cells were treated with a fatty acid (LA, DHA, BSA carrier control) and BSO for 48 h followed by 1 µM 3OH for 24 h. Data represents mean normalized intensity ± SEM of at least 30 images per treatment and 30 cells per image. * indicates significant difference from the corresponding control using two-tailed t-test at p<0.05. Note that all treatments accumulate equally the 3OH and BSO decreased the GSH conjugates which induced higher accumulation of 3OH. In addition, DHA and BSO combined induced the greatest increase in 3OH.

Since DHA treated cells did not show any major changes in BaP metabolites (3OH, t7,8 and Pyr) with GSH or BSO, we evaluated the activity of the glutathionation pathway using a 3OH standard directly as a treatment instead of BaP. BSO significantly increased the 3OH metabolites in all three treatments with the largest increase induced by DHA treated cells (**Figure 8**). This indicates that the gluathionation pathway is highly active in DHA treated cells. This is also verified by measuring the GST activity using monochlorobimane (**Figure E in File S1**) [28]. The fact that no significant results were seen with BSO and BaP in DHA treated cells (**Figure 8**) could indicate the activation of other pathways (such as glucuronidation or sulfation).

### Fatty Acid Effects on Glucuronidation and Sulfation of BaP Metabolites

**Figure 9:**
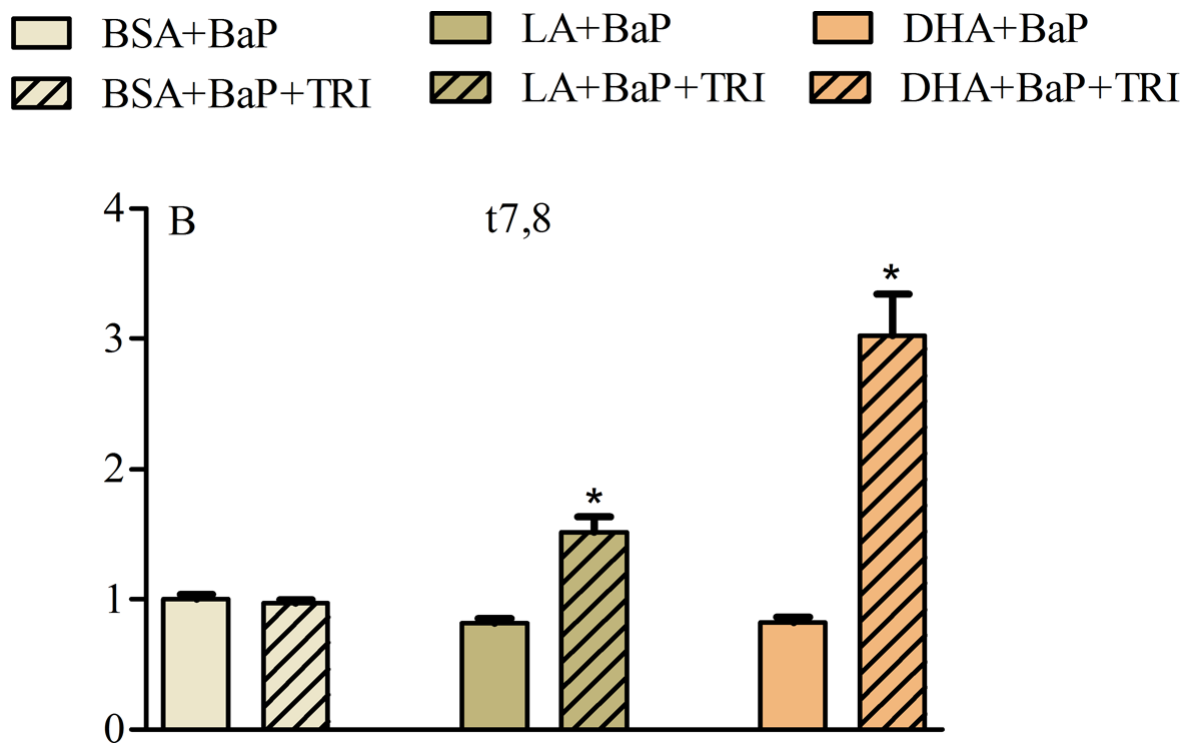
Assessment of BaP metabolites in A549 cells using inhibitors of glucuronidation and sulfation. A) Measurement of 3OH metabolite in A549 cells supplemented with BSA-conjugated fatty acids (LA, DHA, BSA carrier control) for 24 h followed by 2 µM BaP for 24 h in the presence or absence of 500 units of β-glucuronidase. B) Measurement of t7,8 metabolite in A549 cells treated with BSA-conjugated fatty acids (LA, DHA, BSA carrier control) for 24 h followed by 2 µM BaP for 24 h in the presence or absence of 40 µM triclosan. Data represents mean normalized intensity ± SEM of at least 30 images per treatment and 30 cells per image. * indicates significant difference from the corresponding control using two-tailed t-test at p<0.05. Note that glucuronidation (indicated by increase in 3OH due to the presence of β-glucuronidase) is not significant in DHA or LA treated cells while sulfation (indicated by increase in t7,8 due to the presence of triclosan) is significantly enhanced in DHA and LA when compared to BSA.

To investigate the role of fatty acids in the metabolism of BaP through glucuronidation, A549 cells were supplemented with the fatty acids (BSA, LA or DHA) for 48 h followed by BaP and 500 units of β-glucuronidase for 24 h. β-glucuronidase resulted in a modest but significant increase in the free 3OH metabolite of BaP in BSA compared to cells treated with BSA alone whereas LA and DHA treated cells were unaffected by the presence of β-glucuronidase (**Figure 9A**) indicating that 3OH-glucuronidation is not induced in LA or DHA treated cells (Zhu li et al., 2008). In comparison, with respect to glucuronidation of t7,8, no significant glucuronidation was detected in any of the treatments tested (data not shown).

t7,8 is a metabolic precursor of BPDE, the carcinogenic BaP metabolite that alkylates DNA [6]. Formation of t7,8 is dependent on metabolism of BaP into the 7,8 oxide which in turn is hydrolyzed to t7,8. Since t7,8 was significantly reduced in DHA treated cells, we investigated the effect of fatty acids on sulfate conjugation of t7,8 in A549 cells supplemented with either BSA, LA or DHA for 48 h followed by 2 µM BaP in the presence or absence of triclosan (a competitive inhibitor of sulfation and to a lesser extent glucuronidation) for another 24 h. Triclosan added to cells supplemented with BSA did not show any increase in t7,8 metabolite compared to BSA alone (**Figure 9A**). However, triclosan added to cells supplemented with LA or DHA showed a significant increase in t7,8 metabolite compared to the corresponding control (LA alone or DHA alone), with the highest level of t7,8 accumulation present in DHA treated cells (**Figure 9B**).

## Discussion

Metabolism of BaP is complex and involves biological activation through oxidative metabolism by cytochrome p450s and other enzymes responsible for Phase I reactions. The ultimate carcinogen, BPDE, results from metabolic activation by cytochrome P4501A1 and 1B1 enzymes and hydrolysis by epoxide hydrolase [29]. Numerous additional metabolites are also generated including epoxides, phenols, dihydrodiols, quinones, triols, tetrols and diol epoxides [30], [31] and these metabolic products can affect a wide variety of cellular responses. Many of these metabolites can be conjugated to glucuronic acid, sulfate and glutathione in Phase II reactions to become more water soluble facilitating their excretion [7] (**Figure F in File S1**). Real time analysis of the parent compound and selected metabolites has been reported using multiphoton laser scanning microscopy combined with the advanced linear unmixing process in several cell types [13], [32].

Results of the present study which simultaneously examines seven major metabolites (BaP, 3OH, 9OH, t7,8, BPDE, Pyr, 3S and 3,6BPQ) confirm that A549 cells treated with BaP activate CYP1A1/CYP1B1 and produce more reactive intermediates that form DNA adducts (**Figure A in File S1** and **Figure 1**). Supplementing A549 cultured lung cells with fatty acid altered the metabolite distribution primarily through a decrease in 3OH, t7,8 and Pyr-like metabolites (**Figure 3**) with the greatest decrease obtained with DHA supplementation; and this was maintained 24 h after removal of DHA (**Figure 4**). DHA supplementation also decreased BPDE-DNA adduct formation (**Figure 5**). The metabolism of BaP to t7,8 and BPDE is critical for the carcinogenic effects of BaP [2]; and further metabolism of BPDE to tetrols or adducts including DNA results in formation of pyrene derivatives due to loss of aromatization of the D ring of BaP [13]. The observed decrease in BPDE-DNA adducts in cells supplemented with LA or DHA did not result from the decrease in cytochrome P4501A1 and 1B1 enzymes and hydrolysis by epoxide hydrolase since EROD activity was the highest in DHA supplemented cells indicative of increased P450 activity (**Figure 6**). This result is in agreement with the work of Zhou et al. [12] who reported that fish oil rich in EPA and DHA reduces the formation of DNA adducts in B6C3F1 male mice. This decrease in DNA adducts was shown to be correlated with the activation of the phase I enzyme Cyp1a1 and the phase II enzyme Gstt1. Because CYP1A1 has been shown to be involved in both detoxification and metabolic activation in a cell context dependent manner [33]–[35], it is possible that in A549 cells, the increase in EROD activity (increase in CYP1A1/CYP1B1) also contributed significantly to the BaP detoxification. Other factors that contributed to BaP detoxification with LA or DHA supplementation is the increase in sulfation of BaP metabolites in A549 cells. This was verified using triclosan that itself undergoes sulfation and glucuronidation and can be used as a competitive inhibitor of both reactions [20]. In this study, the inhibitory effect of triclosan was investigated as a function of the t7,8 metabolite formed in A549 cells treated with BaP and/or triclosan (**Figure 9B**). This experiment confirmed that sulfation is increased in LA and DHA treated cells with the greatest increase induced with DHA. However, no significant change in glucuronidation was observed in LA or DHA supplemented cells (**Figure 9A**) when two metabolites of BaP (3OH and t7,8) were measured. The selection of these two metabolites was based on the fact that 3OH generates the glucuronidated metabolite directly and t7,8 generates it directly or indirectly. The absence of significant glucuronidation of BaP metabolites (specifically 3OH and t7,8) may be due to the fact that LA and DHA can also be competitively glucuronidated [36] or to the activation of another phase II enzyme such as glutathione S-transferase (**Figure E in File S1**) and therefore the recruitment of other detoxification pathway such as the glutathione pathway.

Glutathionation is important in DHA supplemented cells as A549 cells supplemented with DHA or LA followed by BSO and 3OH exhibited an increase in 3OH accumulation indicative of a decrease in glutathione conjugates (**Figure 8**). These data indicate that DHA treated cells produce less hydroxyl metabolites of BaP possibly due to activation of glutathione S-transferases (**Figure E in File S1**), a class of phase II enzymes, that can detoxify PAHs epoxides, quinones and hydroperoxides by conjugation with glutathione, and/or due to a better export of phase II metabolites in A549 cells [37]. This also may have contributed to the decrease in BPDE-DNA adduct levels. However, when cells were treated with BaP, glutathionation was significant only in control (BSA) cells as determined by inhibition of glutathione-S-transferase (BSO) or addition of GSH and examination of three BaP metabolites (3OH, t7,8 and Pyr-like signals) (**Figure 7**). Therefore other phase II conjugation pathways (such as sulfation) are active (**Figure 9B**).

In addition to the above mentioned changes in BaP metabolites in DHA or LA supplemented cells, a significant increase in the membrane accumulation of the parent compound BaP was observed. This is in agreement with other studies showing that dietary or media lipid changes quickly alter the polyunsaturated fatty acid composition of the membrane phospholipids [38]–[40] and that changes in the fatty acid profiles of the membranes might modify the physicochemical environment of the cell sufficiently to affect such functions as receptor activity, enzyme activity or permeability to chemical agents [41]–[46]. Therefore, the alterations in cell membrane fatty acid composition induced by LA and DHA appear to be factors underlying their differential actions on BaP metabolism [47].

In summary, PUFA (LA and DHA) treatment increased BaP membrane accumulation with the greatest increase induced in DHA supplemented cells. This elevated membrane accumulation in DHA was associated with the induction of phase I (P450) and II (sulfotransferases, glutathione S-transfrases and UDP-glucuronosylransferases) metabolizing and detoxifying enzymes. The overall outcome is the reduction of Pyr-like (adducts including DNA), 3OH and t7,8 metabolites (**Figure 3**) in DHA supplemented cells. Further studies are needed to evaluate development of strategies involving dietary supplement with DHA to reduce the risk of human cancers caused by exposure to environmental PAHs.

## Supporting Information

File S1 **This file includes the following: Figure A. EROD activity in A549 cells.** Cells were treated with different concentrations of BaP for 24 h. Data represent mean fluorescence intensity of resorufin ± SEM of a least 8 replicates per concentration tested. **Figure B. Effect of αNF on BaP metabolites in A549 cells.** Cells were cultured for 24 h in the presence of BSA, treated simultaneously with 100 µM αNF and 2 µM BaP for another 24 h before imaging. Note the reduction in the Pyr and 3S metabolites in cells treated with αNF compared to the corresponding metabolites generated by BaP. *indicates significant difference from the corresponding control metabolite at p<0.05. **Figure C. Effect of resveratrol (R) on BaP metabolites in A549 cells.** Cells were cultured for 24 h in the presence of BSA, treated simultaneously with 20 µM resveratrol and 2 uM BaP for another 24 h before imaging. Note the reduction in parent compound, 9OH and Pyr metabolites in cells treated with resveratrol compared to the corresponding metabolites generated by BaP. *indicates significant difference from the corresponding control metabolite at p<0.05. **Figure D. Short term uptake of BaP in A549 cells.** Note that BSA and DHA treated cells accumulate BaP equally following 1 h exposure. Data represents mean fluorescence intensity ± S.E. of a least 15 images per treatment. **Figure E. GST activity in A549 cells following fatty acids and/or BaP treatments.** A549 cells treated with DHA alone exhibited a slight but significant increase in GST activity when compared to cells treated with BSA alone (A). Cells treated with DHA followed by BaP for 24 h exhibited even a higher increase in GST activity when compared to BSA followed by BaP treatment (B). Data represents GST activitiy (sec^−1^) of at least 50 cells per treatment. * indicates significant difference from the corresponding control at p<0.05. **Figure F. Major metabolic activation pathways of BaP.**
(DOCX)
