# Supplementary figures and images for: Effects of Fatty Acids on Benzo[a]pyrene Uptake and Metabolism in Human Lung Adenocarcinoma A549 Cells
Source: PLoS One. 2014 Mar 20;9(3):e90908. doi: 10.1371/journal.pone.0090908 (PMC3961214; doi:10.1371/journal.pone.0090908)

**
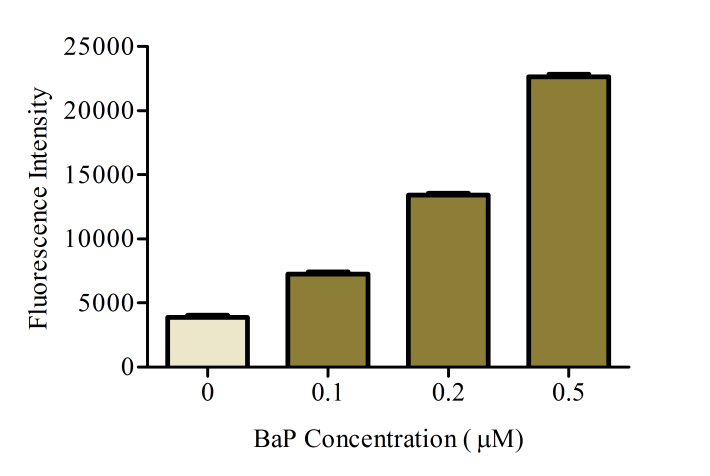
**

**Figure A**


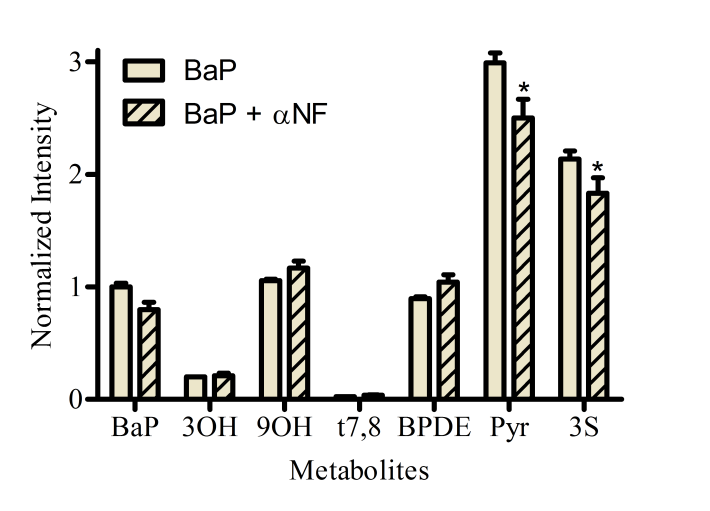


**Figure B**


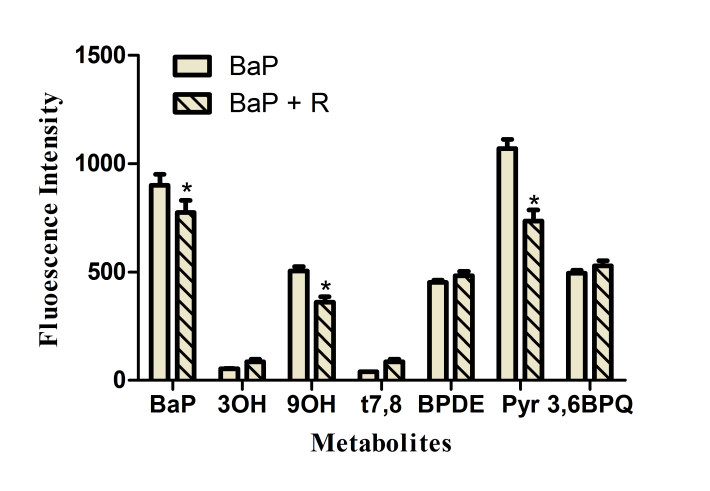


**Figure C**

**
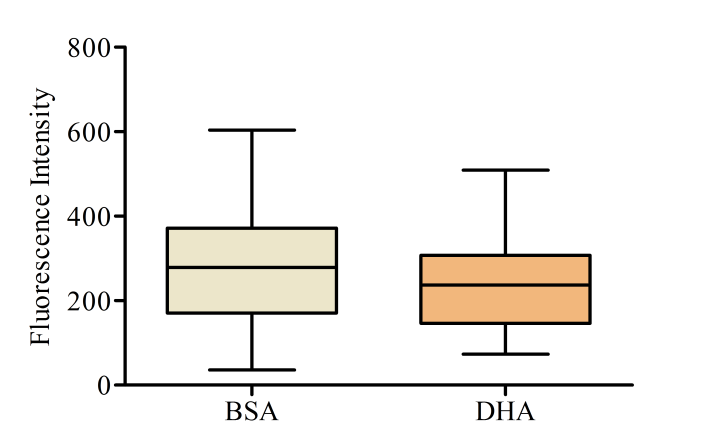
**

**Figure D**


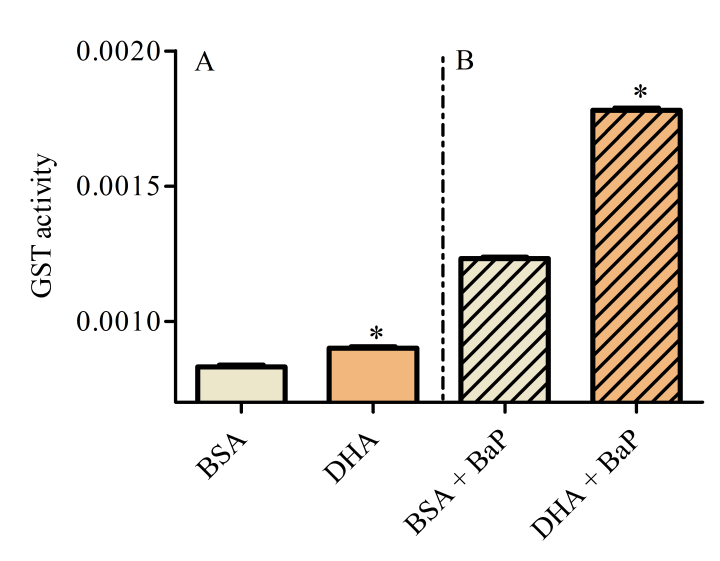


**Figure E**


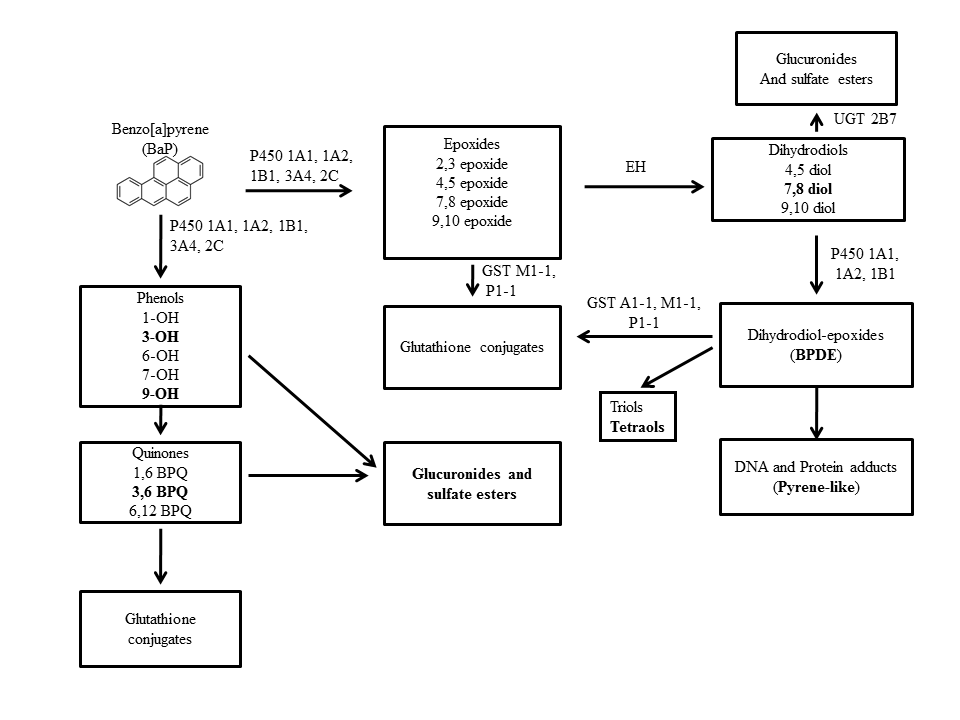


**Figure F**

Supplement: File S1 — This file includes the following: Figure A. EROD activity in A549 cells. Cells were treated with different concentrations of BaP for 24 h. Data represent mean fluorescence intensity of resorufin ± SEM of a least 8 replicates per concentration tested. Figure B. Effect of αNF on BaP metabolites in A549 cells. Cells were cultured for 24 h in the presence of BSA, treated simultaneously with 100 µM αNF and 2 µM BaP for another 24 h before imaging. Note the reduction in the Pyr and 3S metabolites in cells treated with αNF compared to the corresponding metabolites generated by BaP. *indicates significant difference from the corresponding control metabolite at p<0.05. Figure C. Effect of resveratrol (R) on BaP metabolites in A549 cells. Cells were cultured for 24 h in the presence of BSA, treated simultaneously with 20 µM resveratrol and 2 uM BaP for another 24 h before imaging. Note the reduction in parent compound, 9OH and Pyr metabolites in cells treated with resveratrol compared to the corresponding metabolites generated by BaP. *indicates significant difference from the corresponding control metabolite at p<0.05. Figure D. Short term uptake of BaP in A549 cells. Note that BSA and DHA treated cells accumulate BaP equally following 1 h exposure. Data represents mean fluorescence intensity ± S.E. of a least 15 images per treatment. Figure E. GST activity in A549 cells following fatty acids and/or BaP treatments. A549 cells treated with DHA alone exhibited a slight but significant increase in GST activity when compared to cells treated with BSA alone (A). Cells treated with DHA followed by BaP for 24 h exhibited even a higher increase in GST activity when compared to BSA followed by BaP treatment (B). Data represents GST activitiy (sec−1) of at least 50 cells per treatment. * indicates significant difference from the corresponding control at p<0.05. Figure F. Major metabolic activation pathways of BaP. (DOCX) [file pone.0090908.s001.docx]
